# Myostatin and Activin A as Biomarkers of Sarcopenia in Inflammatory Bowel Disease Patients

**DOI:** 10.3390/nu16060810

**Published:** 2024-03-12

**Authors:** Małgorzata Godala, Ewelina Gaszyńska, Konrad Walczak, Ewa Małecka-Wojciesko

**Affiliations:** 1 Department of Nutrition and Epidemiology, Medical University of Lodz, 90-752 Lodz, Poland; ewelina.gaszynska@umed.lodz.pl; 2 Department of Internal Medicine and Nephrodiabetology, Medical University of Lodz, 90-549 Lodz, Poland; konrad.walczak@umed.lodz.pl; 3 Department of Digestive Tract Diseases, Medical University of Lodz, 90-153 Lodz, Poland; ewa.malecka-panas@umed.lodz.pl

**Keywords:** inflammatory bowel disease, sarcopenia, Crohn’s disease, ulcerative colitis, nutritional status, myostatin, activin A

## Abstract

The prevalence of sarcopenia in inflammatory bowel disease patients has received increasing attention. The aim of this study is to assess the usefulness of determining levels of myostatin (MSTN) and activin A (Act A) as potential markers of disease activity and occurrence of sarcopenia in Crohn’s disease and ulcerative colitis patients. The case-control study included 82 patients with Inflammatory Bowel Disease. The control group consisted of 25 healthy volunteers. The serum levels of myostatin and activin A were determined by the quantitative sandwich enzyme-linked immunosorbent assay. Sarcopenia was diagnosed based on the EWGSOP2 criteria. The study found lower levels of myostatin and activin A in the IBD patients. There were significantly lower levels of myostatin (80.6 pg/mL vs. 186.2 pg/mL; *p* = 0.0364) as well as activin A (32.1 pg/mL vs. 35.2 pg/mL; *p* = 0.0132) in the IBD patients with sarcopenia compared to those without sarcopenia. Positive correlations were found between MSTN levels and Muscle Mass Index (rho = 0.31; *p* < 0.005) and hand grip strength (rho = 0.34, *p* < 0.05) in the IBD patients. The determination of serum levels of MSTN and Act A may be useful in the early diagnosis of sarcopenia in IBD patients.

## 1. Introduction

Inflammatory bowel disease (IBD) includes Crohn’s disease (CD) and ulcerative colitis (UC). IBD has an unpredictable clinical course in which periods of remission alternate with acute exacerbations of symptoms [1,2,3]. Patients with IBD suffer from malnutrition, the prevalence of which is estimated at 20–85%, and the causes include insufficient energy intake for fear of exacerbation of symptoms, malabsorption, and chronic inflammation [4,5,6,7,8,9,10]. The prevalence of sarcopenia in these patients has received increasing attention. A meta-analysis conducted in 2018, including five clinical trials and more than 650 IBD patients, showed that 52% of CD patients and 37% of UC patients have sarcopenia [11]. Sarcopenia is a progressive and generalized skeletal muscle disorder associated with an increased risk of adverse consequences which include falls, fractures, physical disability, and even death. Early intervention involving immediate implementation of physiotherapy and supplementation can stop the progression of the disease and even reduce its negative effects. However, today the condition is most often diagnosed at the critical stage, when the patient is already affected by severe functional impairment [6,9,11].

Primary sarcopenia is a disorder exclusively related to the aging process of the body, where muscle mass and strength decrease [9,12]. With age, there is a decline in the contractile properties of muscles and their ability to generate force. Changes can also be observed in the muscle fibers themselves, such as a 50% reduction in available alpha motor neurons and motor units. This causes impaired motor coordination and a decrease in the availability of stem cells that leads to a reduced ability to regenerate muscle fibers [13,14]. Secondary sarcopenia, however, develops due to factors unrelated to the aging process but resulting from the presence of other diseases, chronic conditions, physical inactivity, prevalent inflammation, and increased catabolism [15,16,17,18,19]. Risk factors for secondary sarcopenia include endocrine and neuromuscular disorders, oxidative stress, insulin resistance, or increased fat percentage in the body composition.

The diagnosis of sarcopenia is a problem for modern medicine. The latest guidelines from the European Working Group on Sarcopenia in Older People (EWGSOP) recommend that low muscle strength, which is much easier to assess and is a more reliable parameter than muscle mass for predicting the adverse effects of sarcopenia, should become the main determinant of sarcopenia. Identifying low muscle strength is sufficient to initiate therapeutic intervention and diagnose suspected sarcopenia. Additionally, it has been suggested that to confirm probable sarcopenia, muscle mass testing, focused on its quantity or quality, should be used; and to assess the severity of sarcopenia, physical fitness should be assessed [20,21]. Since sarcopenia can affect quality of life, length of hospitalizations, surgical outcomes, and patient mortality, a procedure aimed at establishing its presence should be a standard part of assessment in IBD patients [5,18,19].

Biochemical markers are still being sought to assess the nutritional status of patients with regard to muscle mass and strength, in order to diagnose sarcopenia at an early stage [22,23,24]. There are also reports on the involvement of myostatin (MSTN) and activin in nutritional status disorders, including sarcopenia [16,18,25,26,27,28,29,30,31]. Myostatin (MSTN), also known as growth differentiation factor 8 (GDF8), and activin A (Act A) belong to the so-called transforming growth factor-β family [32,33]. These proteins are important regulators of cell growth and differentiation in both embryogenesis and the maintenance of homeostasis of the mature body, including the processes of proliferation, differentiation, and apoptosis [34].

MSTN is an inhibitor of muscle growth. Its physiological role is to prevent the hypertrophy of muscle tissue at various stages of the body development. Studies have confirmed that the overexpression of MSTN results in the inhibition of myoblast proliferation and differentiation, while the inhibition of endogenous MSTN results in stimulation of myoblast proliferation and differentiation [35,36]. It has been shown that myoblast cell differentiation leads to the formation of muscle fibers which naturally produce MSTN, and its amount increases as the differentiation process proceeds. After a certain time, in newly formed muscle fibers, an amount of MSTN is produced that inhibits further differentiation, thereby controlling skeletal muscle growth [37]. Moreover, MSTN inhibits the regenerative processes in the skeletal muscles by impairing the activation and proliferation of satellite cells (amphicytes), and the migration of macrophages and myoblasts to the injury site [28]. Studies have shown that mutations in the gene encoding MSTN cause a double increase in skeletal muscle mass in mice, sheep, and cattle [35,36,37,38]. Studies in humans have shown a relationship between high MSTN levels with sarcopenia and reduced survival rates in patients with cirrhosis [34]. It was further proven that an increased MSTN expression in cardiomyocytes caused myocardial interstitial fibrosis, leading to dysfunction of the heart muscle [39], and in a mouse model of heart failure, MSTN released from myocardial cells was associated with skeletal muscle atrophy. Increased myocardial MSTN expression was observed after induced myocardial infarction in eight-week-old mice, and its effects, associated with protein degradation and skeletal muscle atrophy, persisted up to two months after myocardial infarction [40].

Activins were first isolated from the ovarian follicular fluid, and their role was linked to their effect on the regulation and secretion of folliculotropic hormone [41,42,43]. It is now well known that many other organs and tissues are the source of their synthesis, including the pituitary gland, thyroid gland, bone marrow, pancreas, adrenal cortex, liver, and the reproductive organs [41,43]. Studies have demonstrated an involvement of activin A (Act A) in tissue inflammatory and repair responses [44,45,46]. High levels of this protein were observed in cancer patients, especially in cachectic individuals, which implies its association not only with increased inflammation but also with cachexia [47]. A study using experimental animal models showed an association between the development of cancer cachexia and activation of the ActRIIB receptor, a major protein that determines MSTN signaling. Inhibition of ActRIIB resulted in regeneration of mass of the skeletal and cardiac muscles. This finding suggests an important role the activin-dependent signaling pathway plays in inducing muscle cachexia and can be used in the development of therapies against cachexia and sarcopenia [42].

The aim of this study is to assess the usefulness of determining levels of MSTN and Act A as potential markers of disease activity and occurrence of sarcopenia in CD and UC patients.

## 2. Materials and Methods

### 2.1. Study and Control Groups

The case-control study included 82 patients with IBD, with 42 females (51.2%) and 40 males (48.8%) (mean age 38.1 ± 11.6 years), 48 patients with CD, and 34 patients with UC, treated at the Department of Digestive Tract Diseases, Medical University of Lodz. Patients with cancer and cardiovascular and metabolic diseases were excluded from the study. The control group consisted of 25 healthy volunteers. To assess disease activity, the Crohn’s Disease Activity Index (CDAI) and the Montreal classification were applied for patients with CD [48]. For patients with UC, the Partial Mayo Score and the Montreal classification were used [49].

The study was conducted in accordance with the Declaration of Helsinki and approved by the Bioethics Committee of the Medical University of Lodz (No. RNN/70/22/KE). All the subjects gave their written consent to participate in the study. 

### 2.2. Anthropometry

Waist circumference was measured in all the subjects and BMI (Body Mass Index) was calculated. Malnutrition was determined for BMI < 18.5 kg/m^2^, normal nutritional status for BMI from 18.5 to 24.9 kg/m^2^, overweight for BMI from 25.0 to 29.9 kg/m^2^, and obesity from 30.0 kg/m^2^. Also, body composition was measured by electrical bioimpedance using an InBody 270 instrument (Seoul, Republic of Korea). Based on the obtained results, the Muscle Mass Index (MMI) was calculated, expressing the ratio of total skeletal muscle mass to the square of height [kg/m^2^]. The following cutoff points were used to assess significant reduction in muscle mass, i.e., <8.6 kg/m^2^ for males and <6.2 kg/m^2^ for females [20,50].

Hand grip strength (HGS) was measured in all the subjects. Maximum grip strength was measured using a SAEHAN handheld digital dynamometer, model no. DHD-1 (SH1001) (SAEHAN Corporation, Changwon-si, Republic of Korea), with a spring (90 kG). The measurement was performed in a sitting position on a chair without armrests, with the feet resting flat on the floor, the arms along the torso, the elbow joints bent at 90 degrees, the forearm in the neutral position, and the wrist extended at 0–30 degrees. Each subject squeezed the dynamometer bar twice for six seconds, with a one-minute rest break. The better attempt for the dominant hand was considered the relevant measurement [51]. Cut-off points for low muscle strength were adopted according to the EWGSOP2 criteria, i.e., <16 kg for females and <27 kg for males [20].

Sarcopenia was diagnosed based on the EWGSOP2 criteria if both low muscle strength (HGS) and low muscle mass (MMI) occurred [20].

### 2.3. Physical Activity

A short version of the International Physical Activity Questionnaire (IPAQ) was used to assess the level of physical activity. The respondents answered three questions which related to the frequency and duration of engaging in high-, moderate- and low-intensity physical activity that lasted at least ten minutes continuously [52]. To identify intensity zones, examples of typical physical activity were given, representing different levels of intensity. The IPAQ questionnaire defines intense physical activity as heavy exertion which results in strongly increased breathing and accelerated heart rate (e.g., intense aerobics, cycling > 20 km/h, fast swimming, lifting heavy weights, or doing construction works). Moderate physical activity means moderate effort with a slightly accelerated breathing and heart rate (e.g., cycling 10–15 km/h, Nordic walking, jogging, sports games, recreational swimming, downhill skiing on easy trails). Low-intensity exercise is mainly walking [53].

### 2.4. Collection of Blood Samples and Serum Markers of Nutritional Status 

Blood samples for laboratory tests were collected fasting from the basilic vein, then centrifuged (2000× *g* for 20 min). The isolated serum was frozen at −80 °C. The obtained samples were used to determine inflammatory markers.

The serum levels of MSTN and Act A were determined by the quantitative sandwich enzyme-linked immunosorbent assay (ELISA), using kits from Biorbyt LLC, Durham, NC, USA (catalog number orb563298 for MSTN and orb1100986 for Act A). All the tests were performed according to the manufacturer’s instructions. Additional laboratory tests, e.g., peripheral complete blood count, were performed using automatic devices. 

### 2.5. Statistical Analysis

All the computational procedures were performed using Statistica™ 14 (TIBCO Software Inc., Palo Alto, CA, USA). Categorical variables were expressed using integers and percentages. Numerical traits were depicted by means, standard deviations, medians, and lower-to-upper quartiles. The normality of distribution was preliminarily assessed based on the Shapiro–Wilk W test. For univariate analyses, the Mann–Whitney U test was used when a grouping variable was dichotomous, or the Kruskal–Wallis H test was carried out when a grouping variable had more than two categories. Correlations were assessed by Spearman’s coefficient (r). For multivariate procedures, generalized linear models (for non-normally distributed variables) were performed to assess differences in numerical variables between the study groups. All the multivariate models were controlled for the study participants’ age, gender, level of education, family burden, and disease duration. To identify factors associated with sarcopenia, univariate and multivariate analyses were used. To assess the risk of sarcopenia in patients with IBD, receiver operating characteristic (ROC) classification models were used. A value of *p* < 0.05 was considered statistically significant.

## 3. Results

### 3.1. Study Group Characteristics

The characteristics of the study group are presented in Table 1. The groups of patients and healthy subjects were similar in terms of gender, age, BMI, waist circumference, and level of physical activity. Based on BMI in the IBD group, 10 patients (12.2%) were found to be malnourished, 38 patients (46.3%) had normal nutritional status, 26 patients (31.7%) were overweight, and 8 patients (9.8%) were obese. At the time of the study, most of the subjects received biology treatment—infliximab or vedolizumab (*n* = 66, 80.5%). More than half of the participants (*n* = 42, 51.2%) had a low level of physical activity, 25 patients (30.5%) had a sufficient level of physical activity, and 15 individuals (18.3%) had a high level of physical activity.

The IBD patients showed a lower mean HGS (25.4 kG vs. 30.7 kG; *p* < 0.05) and MMI (8.1 kg/m^2^ vs. 10.2 kg/m^2^; *p* < 0.05). Sarcopenia was diagnosed in 21 IBD patients and two controls (25.6% vs. 8%; *p* < 0.05).

### 3.2. Levels of MSTN and Act A

A significantly lower median of MSTN level was found in the CD group compared to the UC group (82 pg/mL vs. 160 pg/mL; *p* = 0.0098). It was also significantly lower than in the control group (430 pg/mL; *p* = 0.002). Significantly lower mean MSTN levels were observed in the UC patients compared to the control group (160 pg/mL vs. 430 pg/mL; *p* = 0.0451).

A significantly lower median of Act A level was found in the CD patients compared to the control group (33.75 pg/mL vs. 36.10 pg/mL, *p* = 0.0113). There were no differences in the median of Act A level between the CD and UC patients (33.75 pg/mL vs. 34.25 pg/mL, *p* = 0.5975). There were no differences in the median of Act A level between the UC patients compared to the control group (34.25 pg/mL vs. 36.10 pg/mL, *p* = 0.1469) (Table 2).

There were no differences in the median of MSTN and Act A levels according to the duration of the disease, previous intestinal resections, or biological treatment (Table 3).

There were no differences in MSTN and Act A levels in the UC patients depending on disease activity as assessed by the Partial Mayo Score and the Montreal classification. Similarly, there were no differences in MSTN and Act A levels in the CD patients depending on disease activity assessed using the Montreal classification and CDAI (Table 4).

### 3.3. Levels of MSTN and Act A and Nutritional Status of IBD Patients

There were significantly higher MSTN levels found in overweight IBD patients compared to those with normal BMI (131.2 pg/mL vs. 88.6 pg/mL; *p* = 0.0227), as well as compared to underweight patients (99.2 pg/mL; *p* = 0.0241). There were no differences in MSTN levels between underweight and well-nourished patients as determined by BMI (Table 5).

There were significantly higher Act A levels in overweight IBD patients compared to those with normal BMI (34.3 pg/mL vs. 31.2 pg/mL; *p* = 0.0192), as well as compared to underweight patients (31.9 pg/mL; *p* = 0.0421). There were no differences in Act A levels between underweight and well-nourished patients as measured by BMI.

There were no differences in MSTN and Act A levels in IBD patients depending on waist circumference, body fat content, and physical activity level.

There were significantly lower levels of MSTN (80.6 pg/mL vs. 186.2 pg/mL; *p* = 0.0364), as well as Act A (32.1 pg/mL vs. 35.2 pg/mL; *p* = 0.0132) in the IBD patients with sarcopenia compared to those without sarcopenia. Additionally, significantly lower levels of MSTN were observed in patients with low MMI compared to those with normal MMI (88.1 pg/mL vs. 219.0 pg/mL; *p* = 0.0317), as well as in patients with low HGS compared to those with normal HGS (64.0 pg/mL vs. 149.7 pg/mL; *p* = 0.0256). Act A levels were significantly lower in patients with low MMI compared to those with normal MMI (31.9 pg/mL vs. 34.2 pg/mL; *p* = 0.0221), as well as in patients with low HGS compared to those with normal HGS (32.0 pg/mL vs. 33.9 pg/mL; *p* = 0.0301).

Positive correlations were found between MSTN levels and MMI (rho = 0.31; *p* < 0.005) and HGS (rho = 0.34, *p* < 0.05) in the IBD patients. Also, there were positive correlations between MSTN and Act A levels in the CD group (rho = 0.628; *p* < 0.0001), the UC group (rho = 0.499; *p* = 0.0026), and all the IBD patients (rho = 0.555; *p* < 0.0001).

### 3.4. Assessment of Sarcopenia Risk in IBD Patients

Levels of MSTN (OR = 0.736, 95% CI 0.439–0.906; *p* = 0.0273) and Act A (OR = 0.665, 95% CI 0.379–0.974; *p* = 0.0229) were shown to be predictors of sarcopenia in the IBD patients after adjusting for age, gender, and BMI of the subjects (Table 6).

**Figure 1:**
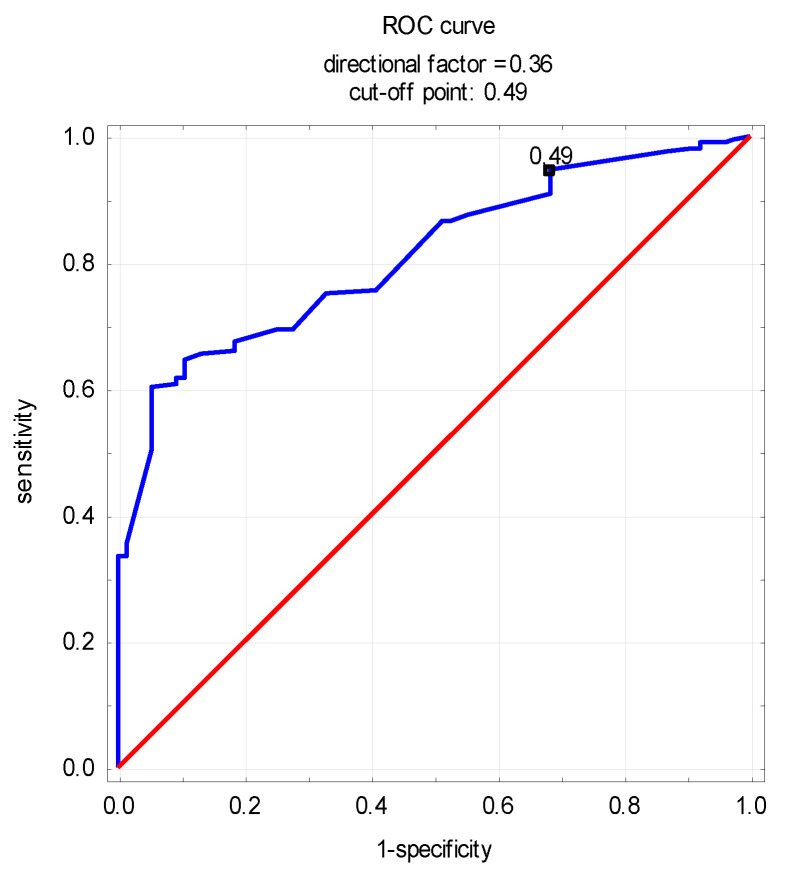
Receiver operating characteristic curve of log_10_myostatin-to-activin A ratio for sarcopenia detection in patients with IBD.

When evaluating the risk of sarcopenia in the IBD patients based on ROC curves, it was not possible to assess the potential occurrence of sarcopenia by determining the levels of MSTN or Act A only. However, the log_10_myostatin-to-activin A ratio was shown to indicate the presence of sarcopenia in this group (cut-off point of 0.49, sensitivity of 93%, specificity of 69%; Area 0.810, *p* < 0.05) (Figure 1).

## 4. Discussion

To the best of our knowledge, this is the first study to assess the serum levels of Act A and MSTN as markers of sarcopenia in IBD patients. There are papers evaluating the prevalence of sarcopenia in IBD patients, as well as works analyzing MSTN and Act A levels in different groups of patients, including those with cardiovascular, metabolic, and neoplastic diseases. There are also studies examining the relationship between Act A levels and chronic inflammation, including in IBD patients. However, no studies were found that evaluated both the relationship between the nutritional status of IBD patients and MSTN and Act A levels as potential markers of sarcopenia.

The role of MSTN as a marker of nutritional status has not yet been clearly established. It is considered to inhibit muscle growth, thus contributing to the development of sarcopenia [29,32,37]. However, its involvement in the development of nutritional status disorders in groups of patients treated for various conditions is still debated, and the results of available studies are contradictory.

In our study, we found significantly lower MSTN levels in the CD and UC patients compared to the healthy subjects. Additionally, we found significantly lower MSTN levels in patients with sarcopenia. In contrast, we found no differences in MSTN levels in the CD and UC patients depending on disease activity.

The available data on the relationship between MSTN level and the occurrence of sarcopenia are inconclusive. Most authors showed an inverse correlation between serum MSTN levels and muscle mass, reporting that higher MSTN levels were associated with decreased muscle mass. Such data were confirmed in studies evaluating the relationship between serum MSTN levels and sarcopenia in patients with cirrhosis and liver cancer [15,34,54,55]. A study by Nishikawa et al. conducted among patients with cirrhosis found that higher serum MSTN levels correlated with loss of muscle mass and impaired protein synthesis [15]. A study by Choi et al. revealed significantly higher serum MSTN levels in patients with liver cancer compared to those observed in healthy subjects. Moreover, in the group of patients, MSTN levels positively correlated with the psoas muscle index (PMI), thus confirming the association of high MSTN levels with decreased muscle mass [34].

The relationship between MSTN and the occurrence of sarcopenia has also been studied in older age groups [10,56,57]. Ryan et al. assessed the serum MSTN level and its possible relationship with sarcopenia among overweight and obese elderly subjects. This study showed significantly higher MSTN levels in patients with sarcopenia compared to those not affected by the condition. The authors of this study also showed a positive correlation between MSTN levels and BMI and body fat of the subjects, thus confirming the relationship between high MSTN levels and muscle atrophy [10]. On the other hand, Chew et al. noted no differences in serum MSTN levels in subjects with and without sarcopenia, so they did not confirm the relationship between high MSTN levels and loss of muscle mass [56]. Similarly, when evaluating MSTN levels in healthy elderly subjects, Peng et al. found that low serum MSTN levels were associated with low skeletal muscle mass in males but not females. These findings cast doubt on the use of the serum MSTN level as a marker of sarcopenia [57].

There are also studies that assess the relationship of MSTN with the occurrence of low muscle mass and sarcopenia in patients with cardiovascular diseases [58,59]. A study by Ishida et al. analyzed MSTN levels in samples of myocardial tissue obtained from patients with advanced heart failure. It showed significantly elevated levels of MSTN in inefficient heart muscles of female patients, but not in male patients. The authors concluded that increased MSTN signaling in females with heart failure may contribute to a higher risk of myocardial infarction and cardiac cachexia [58]. However, in another study, Furihata et al. found reduced serum levels of MSTN in patients with heart failure, compared to healthy subjects. Additionally, serum MSTN levels were associated with muscular atrophy of the lower extremities, which suggests that MSTN may be an important factor in maintaining the mass and strength of skeletal muscles in patients with heart failure [59].

There are also papers found that confirm our own findings. In a study by Alexopoulus et al. conducted in a group of patients with cirrhosis, significantly lower MSTN levels were found in subjects with low muscle mass and sarcopenia, as well as in patients compared to controls. The authors showed that MSTN, together with albumin and creatine kinase, were complementary markers for the assessment of the presence of sarcopenia in these patients [60]. In a study by Yamada et al. conducted in a group of dialysis patients, MSTN levels were positively correlated with the muscle mass of the subjects, indicating significantly lower levels in patients with sarcopenia [61]. A study by Arrieta et al. showed a positive correlation between MSTN levels and lean body mass, as well as greater muscle strength and physical activity. Moreover, MSTN levels were shown to increase after physical exercise had been taken up by the subjects [62]. 

There are several possible reasons for the discrepancies in the data regarding the potential use of MSTN in the assessment of sarcopenia. These ambiguities may be explained by comorbidities that can affect MSTN expression and the entire myokine profile. There are data that confirm the impact of conditions such as insulin resistance, chronic inflammation, cirrhosis, or arteriosclerosis on the increased production of MSTN antagonists such as irisin, follistatin, or IGF-1 [59,63,64,65]. Moreover, these diseases are accompanied by impaired muscle mass and strength, often not yet diagnosed as sarcopenia, but severe enough to result in reduced MSTN production in the muscles. Caution should therefore be exercised when using MSTN as a potential marker of nutritional status in multimorbid patients.

The age of the subjects and their level of training are also important determinants of MSTN production [29,32,66,67,68,69]. In our study, we found no effect of physical activity level on the amount of MSTN concentration. Age did not differentiate MSTN levels either. However, our study group was not very diverse in terms of age or training level. It predominantly included young people, mostly with low to moderate levels of physical activity.

In this study, we also analyzed the relationship of Act A levels with disease activity and nutritional status of the subjects. We found significantly lower Act A levels in the IBD patients compared to the healthy subjects. We did not demonstrate a relationship between Act A levels and IBD activity. However, we recorded lower Act A levels in the patients with sarcopenia compared to those not affected by the condition.

The literature includes papers that assess the relationship of tissue Act A with the pathogenesis of IBD. Act A has been shown to enhance intestinal epithelial migration, as well as to sustain inflammation by contributing to the increased production of pro-inflammatory cytokines [70,71]. The presence of active Act A receptors in the entire gastrointestinal tract was observed in IBD patients, in contrast to healthy subjects. The increased expression of Act A mRNA isolated from the mucosa and submucosa of damaged intestinal epithelium has also been confirmed [71,72,73]. In an animal model, a considerable increase in the fecal concentration of Act A was observed in rats with acute and chronic UC. It was reported that Act A levels in the feces of diseased animals were significantly higher than in healthy animals [74]. However, we could not find papers that assessed Act A levels in the serum of IBD subjects. In our study, we showed a significantly lower level of Act A in the IBD patients compared to the controls. 

There are papers demonstrating the relationship between elevated Act A levels and the occurrence of cardiovascular and metabolic diseases [75,76,77,78,79,80]. A study by Kuo et al. showed higher Act A levels in patients with type 2 diabetes and pre-diabetic conditions, compared to healthy subjects. The authors found that high serum Act A levels were associated with a higher incidence of cardiovascular diseases in these patients [75]. A study by Tsai et al. showed that high serum levels of Act A are associated with a higher risk of the long-term progressive deterioration of renal function in patients after coronary angiography [76]. A study in a group of patients with heart failure reported elevated levels of Act A, concluding that high levels of Act A may contribute to the pathogenesis of myocardial remodeling [77]. A study by Yndestad et al. found significantly higher levels of Act A in hypertensive patients compared to healthy controls. Moreover, high Act A levels were significantly associated with increased mortality in these patients [78].

However, the effect of Act A on muscle tissue has not been clearly described so far. It has been shown that Act A can be secreted by neoplastic cells, which means that it may indirectly be involved in the development of neoplastic cachexia [81,82,83]. A study by Loumaye et al. found higher Act A levels in patients with cancer cachexia than in those not affected by this condition. The study also showed a positive correlation between Act A levels and a decrease in muscle mass [81]. Few papers indicate that Act A contributes to skeletal muscle atrophy observed in animal models of cancer-induced cachexia. Mice, with high levels of circulating Act A, showed loss of skeletal muscle and fat mass which eventually resulted in death [83]. Even in the absence of an underlying disease or cancer, increased levels of local or circulating Act A induce muscle atrophy [83,84,85]. Interestingly, in an animal model of cancer cachexia, the blockade of Act A by a soluble form of its receptor (sActRIIB) prevents muscle atrophy and improves survival, without any effect on tumor growth [83,86]. The relationship between Act A levels and muscular atrophy needs to be confirmed in further analyses.

An interesting result of our study may be the use of MSTN and Act A as markers of sarcopenia in IBD patients. In the present study, with MSTN and Act A levels evaluated separately, it was not possible to establish values that would define patients with or at risk of sarcopenia. However, it was shown that based on the log_10_myostatin-to-activin A ratio, it was possible to divide IBD patients into those with and without sarcopenia at a satisfactory level of sensitivity and specificity.

Our study has some limitations. It is a retrospective, single-center study conducted in a rather small and undifferentiated group of patients. Most of the subjects received biological treatment and had low levels of physical activity. Additionally, the single contact with the subjects made it impossible to assess the influence of factors such as the level of physical activity or therapy applied to the levels of the markers determined. Considering these limitations, further studies are required to confirm the results of the present study.

Patients with IBD are at risk of malnutrition caused by malabsorption, frequent diarrhea, and insufficient dietary intake of energy and nutrients, as shown in studies [87,88,89,90,91]. This also contributes to sarcopenia, the risk of which increases in IBD patients and may be associated with a higher risk of surgery, complications, more frequent hospitalization, and sickness absence. Thus, the search for markers that would improve the early assessment of the risk of sarcopenia in these patients is reasonable. In the present study, the authors demonstrated the potential role of MSTN and Act A in the onset of sarcopenia, while also emphasizing the need to determine both markers. The results of our study suggest that MSTN and Act A appear to be promising markers for the early diagnosis of an abnormal nutritional status and the presence of sarcopenia in IBD. Diagnosing these abnormalities is crucial for implementing proper treatment and rational diet therapy aimed at restoring the normal nutritional status in IBD patients.

## 5. Conclusions

The study found lower levels of MSTN and Act A in the IBD patients, and a further decrease in the concentrations of the two proteins in those with sarcopenia. The determination of serum levels of MSTN and Act A may be useful in the early diagnosis of sarcopenia in IBD patients.

## Figures and Tables

**Table 1:** General characteristics of study participants.

	IBD *n*(%)/Mean ± SD	Controls *n*(%)/Mean ± SD
CD	48 (58.5)	-
UC	34 (41.5)	-
Age [years]	38.1 ± 11.6	38.6 ± 9.1
Female	42 (51.2)	15 (60)
Current smokers	14 (17.1)	3 (12)
Disease duration	8.4 ± 5.7	-
Surgery history	22 (26.8)	-
Disease activity CD		
CDAI [0/1/2/3]	17 (35.4)/10 (20.9)/17 (35.4)/4 (8.3)	-
Montreal classification		
Age at diagnosis [A1/A2/A3]	8 (16.7)/36 (75)/4 (8.3)	-
Disease location [L1/L2/L3]	17 (35.4)/7 (14.6)/24 (50)	-
Disease behavior [B1/B2/B3]	21 (43.8)/18 (37.5) /15 (3.2)	-
Disease activity UC		
Partial Mayo Score [0/1/2/3]	17 (50.0)/0 (0)/11 (32.4)/6 (17.6)	-
Montreal classification		-
Disease location [E1/E2/E3]	4 (11.8)/16 (47.0)/14 (41.2)	-
Severity of relapse [S0/S1/S2/S3]	12 (35.3)/10 (29.4)/11 (32.4)/3 (8.8)	-
Medications		
Infliximab/vedolizumab	66 (80.5)	-
Immunosuppression	33 (40.2)	-
Steroids	25 (30.5)	-
5-ASA	64 (78.0)	-
Anthropometry		
BMI [kg/m^2^]	24.2 ± 4.8	24.6 ± 3.9
<18.5	10 (12.2)	3 (12)
18.5–24.9	38 (46.3)	11 (44)
25.0–29.9	26 (31.7)	9 (36)
>30.0	8 (9.8)	2 (8)
Waist circumference [cm]	88.9 ± 14.5	85.3 ± 9.1
Muscle mass [kg]	43.4 ± 13.7	43.6 ± 9.6
Handgrip strength [kG]	25.4 ± 10.9	30.7 ± 7.5 *
MMI [kg/m^2^]	8.1 ± 4.7	10.2 ± 3.3 *
Sarcopenia [%]	21 (25.6)	2 (8) *
Physical activity		
Low	42 (51.2)	14 (56)
Normal	25 (30.5)	7 (28)
High	15 (18.3)	4 (16)

CD, Crohn’s disease; UC, ulcerative colitis; 5-ASA, 5-aminosalicylic acid; BMI, body mass index; MMI, muscle mass index; * *p* < 0.05.

**Table 2:** Concentrations of myostatin and activin A in the study participants.

		Statistical Parameter					*p*-Value *
		Mean	SD	Me	Q1–Q3	Min.–Max.	
Myostatin [pg/mL]	CD	284	416	82	42–405	12–1680	<0.0001 **
	UC	807	1094	160	69–1119	17–3631	
	Control	1252	1420	430	365–2051	309–4157	
Activin A [pg/mL]	CD	59.06	61.34	33.75	31.55–47.20	28.30–313.20	=0.0231 **
	UC	93.61	212.26	34.25	32.30–39.90	38.60–1173.60	
	Control	160.48	159.02	36.10	32.30–368.200	29.30–400.70	

SD, standard deviation; Me, median; Q1, first quartile; Q3, third quartile; * statistical significance for the model used. All between-group comparisons were controlled for the patients’ age and gender; ** post-hoc multiple comparisons. Myostatin, CD vs. UC *p* = 0.0098; CD vs. controls *p* = 0.0002; UC vs. controls, *p* = 0.0451; Activin A, CD vs. UC *p* = 0.5975; UC vs. controls *p* = 0.1469; CD vs. controls *p* = 0.0113.

**Table 3:** Concentrations of myostatin and activin A according to basic clinical characteristics of patients with IBD.

	Myostatin [pg/mL] Me [Q1–Q3]	Activin A [pg/mL] Me [Q1–Q3]
**Disease duration**		
<5	95.9 [56.8–200.8]	34.4 [31.9–39.5]
5–10	120.8 [36.2–695.8]	33.6 [32.1–37.4]
>10	84.9 [53.4–1630.8]	33.6 [31.9–54.2]
*p*-value	0.6439	0.8086
**Biology therapy**		
YES	93.2 [49.5–672.0]	30.8 [29.0–35.6]
NO	101.8 [50.9–560.6]	33.1 [31.9–38.8]
*p*-value	0.9860	0.4944
**Surgery history**		
YES	54.9 [45.1–200.8]	32.7 [31.9–36.6]
NO	119.9 [64.8–767.6]	34.5 [32.2–42.9]
*p*-value	0.1140	0.2717

Me, median; Q1, first quartile; Q3, third quartile.

**Table 4:** Concentrations of myostatin and activin A depending on UC and CD activity.

	Myostatin [pg/mL] Me [Q1–Q3]	Activin A [pg/mL] Me [Q1–Q3]
UC patients		
Partial Mayo Score		
0	417.8 [52.5–1119.2]	37.9 [29.9–46.3]
2	450.8 [69.4–2432.6]	32.9 [32.3–36.9]
3	98.7 [76.9–311.0]	34.2 [32.4–36.4]
*p*-value	0.8862	0.6958
Montreal Classification		
E1	1367.5 [276.8–2368.3]	31.1 [29.7–290.4]
E2	102.6 [69.4–1119.2]	37.9 [32.3–43.2]
E3	119.5 [71.2–898.0]	33.6 [32.7–36.9]
*p*-value	0.7418	0.4253
S0	417.8 [44.1–2233.9]	37.9 [33.3–173.1]
S1	501.2 [77.9–1119.2]	32.3 [31.5–39.9]
S2	99.8 [69.4–1265.1]	35.8 [32.7–39.5]
S3	183.9 [56.8–311.0]	34.2 [33.6–34.9]
*p*-value	0.7625	0.3358
CD patients		
Age at onset (years)		
A1 < 16	84.2 [50.4–151.0]	37.0 [34.2–51.6]
A2 17–40	84.9 [42.1–656.2]	33.5 [30.9–50.8]
A3 > 40	44.5 [33.7–665.8]	32.8 [31.1–138.8]
*p*-value	0.6266	0.3525
Localization		
L1 Ileum	77.9 [36.2–171.8]	33.7 [30.9–51.1]
L2 Colon	319.2 [67.5–1016.1]	33.1 [32.3–115.8]
L3 Ileum + colon	78.3 [40.5–179.9]	34.4 [31.9–43.6]
*p*-value	0.2964	0.9434
Course of the disease		
B1 No stenoses or fistulas	71.0 [41.3–672.0]	33.8 [30.9–115.8]
B2 Stenoses	61.7 [35.1–120.3]	32.4 [31.5–36.6]
B3 Fistulas	71.9 [42.1–174.2]	34.4 [32.0–35.5]
Perianal lesions	99.9 [58.7–572.0]	32.5 [30.5–50.8]
*p*-value	0.1319	0.9293
CDAI		
<150	84.9 [53.4–672.0]	35.1 [32.6–115.8]
150–220	67.5 [32.8–174.2]	32.6 [31.5–36.6]
221–450	78.3 [40.5–179.9]	34.4 [30.8–37.5]
>450	95.9 [55.9–176.8]	47.1 [30.3–122.8]
*p*-value	0.8282	0.1216

Me, median; Q1, first quartile; Q3, third quartile.

**Table 5:** Concentrations of myostatin and activin A according to nutritional status of IBD patients.

	Myostatin [pg/mL] Me [Q1–Q3]	Activin A [pg/mL] Me [Q1–Q3]
**BMI**		
<18.5	99.2 [52.5–372.0]	31.9 [29.1–37.5]
18.5–24.9	88.6 [49.5–482.0]	31.2 [30.9–46.3]
>25	131.2 [99.5–350.2]	34.3 [31.9–39.5]
*p*-value	0.0369 *	0.0132 *
**Waist circumference**		
Normal	102.6 [52.5–695.8]	33.9 [31.9–39.9]
High	71.9 [44.1–656.2]	33.6 [32.0–54.2]
*p*-value	0.5437	0.6571
**Fatty tissue**		
Low	31.1 [14.9–280.9]	35.1 [32.5–39.7]
Normal	20.2 [9.6–77.5]	32.3 [30.5–39.9]
High	17.1 [10.4–109.3]	33.5 [32.0–46.3]
*p*-value	0.2525	0.2674
**Sarcopenia**		
Yes	80.6 [42.1–179.9]	32.1 [29.0–33.6]
No	186.2 [69.4–890.0]	35.2 [32.9–36.9]
*p*-value	0.0364	0.0132
**MMI**		
Low	88.1 [50.2–220]	31.9 [29.1–33.1]
Normal	219 [69.3–595.2]	34.2 [32.5–36.5]
*p*-value	0.0317	0.0221
**HGS**		
Low	64.0 [40.0–256.1]	32.0 [28.9–33.1]
Normal	149.7 [69.4–383.9]	33.9 [31.8–35.9]
*p*-value	0.0256	0.0301
**Physical activity**		
Low	90.6 [69.4–417.8]	34.3 [32.2–37.1]
Normal	99.2 [35.6–485.4]	33.3 [30.9–45.3]
High	78.3 [53.4–1119.2]	33.0 [31.7–36.9]
*p*-value	0.4678	0.4487

Me, median; Q1, first quartile; Q3, third quartile; * univariate analyses. Post-hoc multiple comparisons. Myostatin, BMI < 18.5 vs. 18.5–24.9, *p* = 0.1221; <18.5 vs. >25, *p* = 0.0241; 18.5–24.9 vs. >25, *p* = 0.0227. Activin A, BMI < 18.5 vs. 18.5–24.9, *p* = 0.3211; <18.5 vs. >25, *p* = 0.0421; 18.5–24.9 vs. >25, *p* = 0.0192.

**Table 6:** Univariate and multivariate analysis to identify factors associated with the presence of sarcopenia in IBD patients.

Characteristic	Univariate Analysis			Multivariate Analysis		
	Odds Ratio	95% CI	*p* Value	Odds Ratio	95% CI	*p* Value
Age	0.564	0.334–1.974	0.5567			0.7761
Sex	0.441	0.398–1.045	0.4421			0.6589
BMI	0.789	0.438–0.991	0.0187			0.0491
Myostatin	0.881	0.412–0.997	0.0221	0.736	0.439–0.906	0.0273
Activin A	0.784	0.541–1.112	0.0302	0.665	0.379–0.974	0.0229

## Data Availability

The data presented in this study are available on request from the corresponding author. The data are not publicly available due to privacy.
